# Multifunctional Benzoxazines Feature Low Polymerization Temperature and Diverse Polymer Structures

**DOI:** 10.3390/polym8080278

**Published:** 2016-08-02

**Authors:** Marc Soto, Matthias Hiller, Hartmut Oschkinat, Katharina Koschek

**Affiliations:** 1 Adhesive Bonding Technology and Surfaces, Fraunhofer Institute for Manufacturing Technology and Advanced Materials IFAM, Wiener Strasse 12, Bremen 28359, Germany; marc.soto@ifam.fraunhofer.de; 2 NMR-supported Structural Biology, Leibniz-Institut für Molekulare Pharmakologie, Robert-Rössle-Strasse 10, Berlin 13125, Germany; hiller@fmp-berlin.de (M.H); oschkinat@fmp-berlin.de (H.O)

**Keywords:** benzoxazine, polybenzoxazine, thermosets, ring-opening polymerization

## Abstract

3,4-dihydro-3-phenyl-2H-1,3-benzoxazines derived from phenol-, resorcinol-, and phloroglucinol give monomers with one, two, and three oxazine units at a single benzene ring, respectively. Aside from the synthesis and characterization of such multifunctional benzoxazines, reactivity and polymerization behavior is studied in dependence of the oxazine functionality. Monomer reactivities are directly related to the number of oxazine functionalities present at the benzene ring yielding the lowest polymerization temperature for the trifunctional phloroglucinol-based benzoxazine. Comparing the polymerization processes and resulting structures, the trifunctional benzoxazine derivative enter new polymerization pathways, which include methylene linkages bridging aniline units, as well as the formation of carbonyl-derived structures.

## 1. Introduction

Materials that are ideally suited to structural applications must be easy to manufacture, exhibit a long shelf life, and preserve their mechanical properties over a wide range of temperatures, especially when these materials are intended for high-end applications, such as in the aeronautical, automotive, or microelectronic industries.

Phenolic thermosetting resin products meet some of these requirements, as they exhibit low flammability, low smoke generation, and good thermal and electrical properties. However, they require strong reagents (acids for novolac and bases for resole resins), and they release water during the curing process, generating voids. Furthermore, brittleness and a limited shelf life restrict the applicability of phenolic-based materials [1,2].

Thermosetting polybenzoxazines have the potential to compete with phenolic resins. The advantages of polybenzoxazines are their mechanical strength, good thermal properties, low water absorption, high glass transition temperatures, and high char yields. Additionally, they polymerize without the need for initiators, do not release byproducts, and display near-zero shrinkage [2,3,4]. Their high curing temperatures, however, are currently a limiting factor in the application of polybenzoxazines [3,5].

**Scheme 1:**
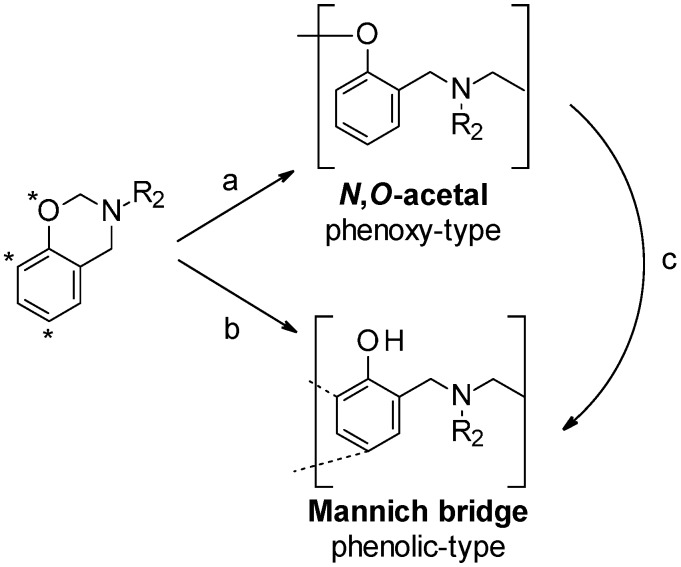
Benzoxazine and polybenzoxazine structures. Nucleophilic attack by oxygen (**a**) or aromatic carbon (**b**) and rearrangement (**c**).

Generally, the polymerization of 3,4-dihydro-1,3-benzoxazines can yield two types of polymer structures depending on the atom that is performing the nucleophilic attack on the electrophilic methylene group (NCH_2_O). An attack by phenolic oxygen initially yields N,O-acetal linkages (phenoxy-type structure), which can then rearrange into Mannich-type structures exhibiting free phenolic units (phenol-type structure). In contrast, an attack by a phenolic carbon (ortho and para positions in benzoxazine) directly results in phenolic-type structures (Scheme 1). Intra- and intermolecular hydrogen bonding of phenolic units with other groups, such as amine, ether, alcohol groups or aromatic rings, is a key factor in the performance of polybenzoxazines. Polymers derived from *N*-methylbenzoxazines tend to form intramolecular hydrogen bonds, while, polymers based on *N*-phenylbenzoxazines form preferably hydrogen bonds between chains. These interactions between different polymer chains improve the performance and mechanical properties of polybenzoxazines based on aromatic amines, making them more interesting for structural applications [6,7].

The main driving force for ring-opening polymerization (ROP) is the ring stress relief caused by the opening of the distorted 6-membered oxazine ring. The ROP of benzoxazines require significantly higher temperatures compared to epoxy rings as benzoxazine ring strain is considerably lower. To overcome this limitation, research efforts have concentrated mainly on two approaches: the use of catalysts and initiators, and the design of highly reactive benzoxazines.

Ring-opening polymerizations of benzoxazines have been conducted using protic compounds (e.g., carboxylic acids [8], phenols [9], thiols [10]), Lewis acids (e.g., phosphorus pentachloride, titanium chloride, aluminum chloride [11], boron trifluoride [12], lithium iodide [13,14]), bases (e.g., imidazole [15]), and latent thermoinitiators (e.g., benzyl tetrahydrothiophenium hexafluoroantimonate [16]). The use of initiators results in lower curing temperatures; however, it also decreases benzoxazine/initiator mixture shelf life [16] and influences the polymer structure [17].

The other approach concentrates on the preparation of highly-reactive benzoxazine monomers. Studies on the electronic effects of phenol-based *N*-phenylbenzoxazines have revealed that electron-withdrawing substituents on the benzene ring at the para position to the oxygen lowered curing temperatures when compared to unsubstituted *N*-phenylbenzoxazine (onset curing temperature *T*_c_ = 263 °C). In contrast, electron-withdrawing groups on the aniline ring in the para position increased the curing temperatures of benzoxazines [1,18].

Especially interesting is the case of resorcinol-based *N*-phenylbenzoxazines, in which two oxazine rings influence each other in the same phenolic ring. These resorcinol-based *N*-phenylbenzoxazines exhibit a low curing temperature (*T*_max_ = 179 °C) [16,19]. This reported neighboring effect has led us to question whether this feature could be extrapolated to a multi-annulated compound exhibiting two neighboring benzoxazine rings.

Herein, we report on a comparison between phenol-, resorcinol-, and phloroglucinol-based *N*-phenylbenzoxazines with one, two and three benzoxazine units in the same phenolic ring, respectively. We have investigated thermal ring-opening polymerization in order to find a direct relationship between reactivity and the degree of substitution. Furthermore, we have gained insights into the polymerization mechanisms and the resulting structures.

## 2. Experimental Section

### 2.1. Materials

Phenol, resorcinol, phloroglucinol, aniline, and paraformaldehyde were purchased from Sigma-Aldrich (Steinheim, Germany) and used as received.

### 2.2. Characterization and Measurements

Infrared spectra (IR) were recorded with a Bruker Equinox 55 FTIR-spectrometer (Bruker Optic GmbH, Ettlingen, Germany) equipped with an attenuated total reflection (ATR) Golden Gate cell at 22 ± 1 °C with a resolution of 4 cm^−1^ in the wavenumber range 600–4000 cm^−1^, and the signal averaged over 32 scans. For data evaluation, the software OPUS 5.5 (Bruker Optic GmbH, Ettlingen, Germany) was applied.

^1^H NMR and ^13^C NMR spectra were carried out at room temperature (rt) with a Bruker Avance NB 200 MHz (Bruker, Rheinstetten, Germany). Chemical shifts (δ) were reported in parts per million (ppm) relative to tetramethylsilane (TMS) using the solvent residual peak as reference. The ^1^H NMR spectra were taken at 200 MHz with 32–64 scans accumulated and ^13^C NMR spectra were taken at 50 MHz with 512 scans accumulated.

Differential scanning calorimetry (DSC) were carried out in aluminum sealed pans with a modulated thermal analyzer DSC 2920 CE (TA Instruments, Hüllhorst, Germany) using N_2_ as a flowing gas over a temperature range from 20 to 300 °C.

Thermogravimetric analysis (TGA) were carried out with a Q5000 TGA (TA Instruments, Hüllhorst, Germany) over a temperature range from 20–800 °C and a heating rate of 10 K/min.

Solid-state magic angle spinning (MAS) NMR data were collected on a Bruker Avance spectrometer (Bruker Biospin, Karlsruhe, Germany) with a 14.09 T magnet, operating at ^1^H and ^13^C Larmor frequencies of 600.13 and 150.903 MHz, respectively. A 4 mm double-resonance (^1^H, ^13^C) MAS probe using a MAS frequency of 6.666 kHz and a VT-set temperature of 290 K (corresponding to 294 K sample temperature) was used. All ^13^C spectra were indirectly referenced to the ^1^H chemical shift of sodium-3-(trimethylsilyl)propanesulfonate (DSS) using a ^13^C chemical ratio of 25.1449530. The recycle delay of a ^1^H–^13^C cross polarization (^13^C CP) experiments is dependent on the ^1^H T_1_-relaxation times [20]. Therefore, ^1^H-T_1_ time measurements were done using the inversion recovery pulse sequence, with a recycle delay of 20 s and a proton π or π/2 pulse length of 5 or 2.5 μs, respectively (Table 1).

The ^13^C CP experiments were done using the following experimental conditions optimized with unlabeled glycine: (1) ^1^H excitation pulse 2.5 μs; (2) magnetization transfer from ^1^H to ^13^C via a ramped CP with a contact time of 2 ms. Spinlock field strength of 56 and 72 kHz were used for ^1^H and ^13^C, respectively; (3) during acquisition proton decoupling using SPINAL 64 with a proton RF field of 90 kHz were applied [21]. The recycle delay (D1) in each experiment were set to 1.3x times the ^1^H T_1_-time; and (4) each experiment was recorded with 1024 scans. Additionally, spectra were recorded under the same conditions using the Total Sideband Suppression (TOSS) sequence to suppress spinning side bands in the spectra [22,23].

### 2.3. Synthesis of N-Phenyl-2H-1,3-Benzoxazines

#### 2.3.1. Synthesis of 3-Phenyl-3,4-Dihydro-1,3-Benzoxazine (P-a)

P-a was synthesized as previously reported [1,24]. Paraformaldehyde (3.60 g, 120 mmol) was dissolved in hot toluene (200 mL). After the addition of aniline (5.5 mL, 60 mmol) dissolved in toluene (10 mL), the reaction was stirred under reflux for 30 min. Phenol (5.6 g, 60 mmol) in toluene (40 mL) was added one drop at a time and the mixture was stirred under reflux for a further 18 h. The reaction was cooled down to rt, and the solvent was evaporated under reduced pressure. The resulting pale yellow solid was dissolved in diethyl ether (100 mL) and washed with an aqueous basic solution (NaOH 1 M, 3 × 100 mL) and water. The organic fraction was dried with anhydrous NaSO_4_ and the solvent was removed under reduced pressure. Recrystallization from hot hexane produced a pale yellow product (6.5 g, 51%).

Melting point (mp) (DSC: onset 5 K/min): 49.3 °C. ^1^H NMR (200 MHz, DMSO-d_6_, ppm): δ 4.65 (s, 2H, NC*H*_2_C_ar_), 5.44 (s, 2H, NC*H*_2_O), 6.72 (d, *J*_3_ = 7.8 Hz, 1H, C_para_*H*), 6.85 (t, *J*_3_ = 7.8 Hz, 2H, C_meta_*H*), 7.04–7.28 (complex absorption, 6H, C_ar_*H*). IR (ATR, cm^–1^): 1599, 1491, and 1454 (aromatic skeletal vibration), 1225 (asymmetric stretching C–O–C_ar_), 1032 (symmetric stretching C–O–C_ar_), 932 (out-of-plane vibration, benzene ring with oxazine), 777 (out-of-plane oxazine vibration), and 752 (stretching N–C_ar_). ^1^H NMR, IR and DSC of P-a can be found in supporting information, Figure S1, Figure S2 and Figure S3 show, respectively.

#### 2.3.2. Synthesis of 3,9-Diphenyl-3,4,9,10-Tetrahydro-[1,3]Oxazino[6,5-f][1,3]Benzoxazine (R-a)

The synthesis was performed adapting described protocols [16,19]. Aniline (10.9 mL, 120 mmol) and paraformaldehyde (7.2 g, 240 mmol) were mixed and stirred in ethyl acetate (100 mL) for 60 min under reflux. A solution of resorcinol (6.6 g, 60 mmol) in ethyl acetate was added, and the mixture was allowed to react for a further six hours. The reaction was cooled and washed with water (3 × 100 mL). After that, the organic layer was dried with anhydrous NaSO_4_ and concentrated with a nitrogen stream. Through cooling the solution in an ice/brine bath, the product was precipitated and then collected by filtration (9.0 g, 44%).

mp (DSC: onset 5 K/min): 136.4 °C. ^1^H NMR (200 MHz, DMSO-d_6_, δ, ppm): 4.43 (s, 2H, NC*H*_2_C_Ar_), 4.56 (s, 2H, NC*H*_2_C_ar_), 5.34 (s, 2H, NC*H*_2_O), 5.49(s, 2H, NC*H*_2_O), 6.30 (d, *J*_3_ = 8.4 Hz, 1H, C_Ar_*H*), 6.81–6.89 (complex absorption, 3H, C_Ar_*H*), 7.04–7.28 (complex absorption, 8H, C_Ar_*H*). IR (ATR, cm^–1^): 1589 and 1487 (aromatic skeletal vibration), 1248 (asymmetric stretching C–O–C_ar_), 1219, 1194, 1038 (symmetric stretching C–O–C_ar_), 928 (out-of-plane vibration, benzene ring with oxazine), 795 and 781 (out-of-plane oxazine vibration), and 752 (stretching N–C_ar_). ^1^H NMR, IR and DSC of R-a can be found in Figure S4, Figure S5 and Figure S6 respectively.

#### 2.3.3. Synthesis of 3,7,11-Triphenyl-3,4,7,8,11,12-Hexahydro-2*H*-1,5,9-Trioxa-3,7,11-Triazatriphenylene (T-a)

Following a method already described [25], paraformaldehyde (7.2 g, 240 mmol) was dissolved in a mixture of ethanol (100 mL) and aqueous sodium hydroxide (7.5 mL, 1 M NaOH). Upon complete solution, aniline was added (10.9 mL, 120 mmol) and stirred for 30 min. The reaction was acidified (15 mL, 1 M HCl) and cooled with an ice/brine bath before the slow addition of a solution of 1,3,5-trihydroxybenzene (5.0 g, 40 mmol) in ethanol (50 mL). After 30 min, the yellow precipitate was separated by filtration, washed with cold ethanol, and dried under vacuum to yield the final product as a yellow solid (14.8 g, 78%).

^1^H NMR (200 MHz, DMSO-d_6_, δ, ppm): δ 4.37 (s, 6H, C_ar_C*H*_2_N), 5.40 (s, 6H, NC*H*_2_O), 6.86 (t, *J*_3_ = 7.6 Hz, 3H, C_para_*H* ), 7.07 (d, *J*_3_ = 7.6 Hz, 6H, C_ortho_*H*), 7.24 (t, *J*_3_ = 7.6 Hz, 6H, C_meta_*H*). ^13^C NMR (50 MHz, DMSO-d_6_, ppm): δ 44.6 (C_ar_*C*H_2_N), 78.8 (N*C*H_2_O), 100.6 (*C*_ar_CH_2_), 117.5 (aniline, *C*_meta_), 120.6 (aniline, *C*_para_), 129.2 (aniline, *C*_ortho_), 147.9 (aniline, *C*_ipso_), 149.6 (*C*_ar_O). IR (ATR, cm^–1^): 1599 and 1495 (aromatic skeletal vibration), 1250 (asymmetric stretching C–O–C_ar_), 1040 (symmetric stretching C–O–C_ar_), 930 (out-of-plane vibration, benzene ring with oxazine), 795 and 781 (out-of-plane oxazine vibration), and 752 (stretching NC_ar_). ^1^H NMR, ^13^C NMR, IR and DSC of T-a can be found in Figure S7, Figure S8, Figure S9 and Figure S10, respectively.

### 2.4. Polymerization Process

The appropriate *N*-phenylbenzoxazine monomers were placed in a silicon mold and the following temperature program was applied: 110 °C for 60 min; 140 °C for 30 min; 160 °C for 30 min; 170 °C for 45 min; 180 °C for 45 min; 190 °C for 60 min; and 200 °C for 90 min.

Poly(P-a): Transparent red solid. IR (ATR, cm^–1^): 3362 (stretching C_ar_O–H), 1599, 1499, and 1456 (aromatic skeletal vibration), 1306 (stretching C_ar_–OH), and 752 (stretching N–C_ar_).

Poly(R-a): Red solid. IR (ATR, cm^–1^): 3372 (stretching C_ar_O–H), 1578 and 1497 (aromatic skeletal vibration), 1292 (stretching C_ar_–OH), and 752 (stretching N–C_ar_).

Poly(T-a): Dark red-brown solid. IR (ATR, cm^–1^): 3377 (stretching C_ar_O–H), 1580, 1501, and 1447 (aromatic skeletal vibration), 1294 (stretching C_ar_–OH), and 752 (stretching N–C_ar_).

IR spectra of poly(P-a), poly(R-a) and poly(T-a) can be found in Figure S11, Figure S12 and Figure S13, respectively. ^13^C CP-TOSS of poly(P-a), poly(R-a) and poly(T-a) can be found in Figure S14, Figure S15 and Figure S16, respectively.

### 2.5. Reaction of T-a with N,N-Dimethylaniline

Benzoxazine T-a (0.055 g, 0.11 mmol) and *N*,*N*-dimethylaniline (0.081 g, 0.67 mmol) were mixed and stirred at 120 °C for 60 min. An aliquot was dissolved in DMSO-*d*_6_ and the soluble part analyzed by ^1^H NMR. *N,N*-dimethylaniline and 4,4′-methylenebis-*N*,*N*-dimethylaniline [26] were observed in a ratio ca. 1:2.

^1^H NMR (200 MHz, DMSO-*d*_6_, δ, ppm): 2.82 (s, 12H, C*H*_3_), 3.68 (s, 2H, C*H*_2_), 6.64 (d, *J*_3_ = 8.7 Hz, 4H, C_meta_*H*), and 7.00 (d, *J*_3_ = 8.7 Hz, 4H, C_ortho_*H*).

Analogous procedures were performed with P-a and R-a. With P-a, the ^1^H NMR of reaction showed a mixture of unreacted reagents (benzoxazine P-a and *N*,*N*-dimethylaniline). In the reaction using R-a, only *N*,*N*-dimethylaniline was observed. No 4,4'-methylenebis-*N,N*-dimethylaniline was observed in either cases.

## 3. Results and Discussion

**Figure 1:**
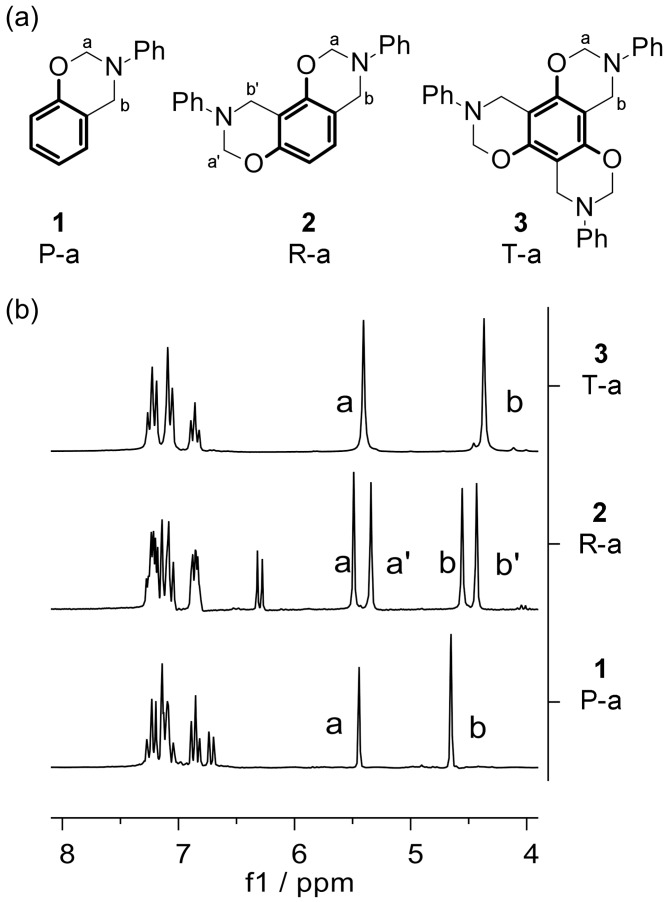
(**a**) *N*-phenylbenzoxazines based on phenol, resorcinol, and phloroglucinol (1,3,5-trihydroxybenzene). “a” *N*,*O*-acetal methylene groups. “b” N*CH*_2_C_ar_ units; (**b**) ^1^H NMR of P-a, R-a, and T-a.

Benzoxazine nomenclature is based on a naming convention that uses capital letters for the phenol component and lower case letters for the amine moiety [2]. Thus, phenol-based and resorcinol-based *N*-phenylbenzoxazines, both bearing aniline as the amine moiety, have been abbreviated to P-a and R-a, respectively. In this paper, phloroglucinol-based *N*-phenylbenzoxazine has been designated T-a, based on its systematic name 1,3,5-trihydroxybenzene, in order to avoid confusion (Figure 1a).

### 3.1. Benzoxazine Syntheses

3,4-Dihydro-3-phenyl-2*H*-1,3-benzoxazines can be seen as the condensation of phenol and aniline with formaldehyde [2]. Accordingly, P-a was obtained through a reaction of phenol, aniline, and paraformaldehyde in toluene. As expected, the ^1^H NMR spectrum of this monofunctional benzoxazine presented two methylene singlets: one corresponding to *N*,*O*-acetal (DMSO-d_6_, 5,44 (a)) and a second one corresponding to C_ar_C*H*_2_N (DMSO-d_6_, 4.65 (b)) (P-a, Figure 1b) [1,24].

The synthesis of R-a, the *N*-phenylbenzoxazine based on resorcinol, can potentially yield two different isomers: one symmetric and another asymmetric. Recently, Schäfer et al. [19] have reported the preparation of the single asymmetric isomer by slow solvent evaporation after the reaction of resorcinol, aniline, and formalin in ethyl acetate. Through an adaptation of this procedure, R-a was prepared by the reaction of resorcinol, aniline, and paraformaldehyde, avoiding the use of formalin. The ^1^H NMR of our reaction product showed two NC*H*_2_O signals (DMSO-d_6_, 5.34 (a’), 5.49 (a)) and two C_ar_C*H*_2_N signals (DMSO-d_6_, 4.43 (b’), 4.56 (b)) with the same integral (R-a, Figure 1b) [16,24]. This result confirmed that, exclusively, the asymmetric isomer of R-a was obtained, as the symmetric isomer would have shown only one signal for each type of methylene group.

T-a was prepared from phloroglucinol (1,3,5-trihydroxybenzene), aniline, and paraformaldehyde. There are two factors of relevance for a successful synthesis: a low temperature (0 °C) and the order of reagent addition. The simultaneous presence of phloroglucinol and formaldehyde in the reaction mixture can lead to a fast formation of an insoluble phenol/formaldehyde-type resin. To avoid this, aniline was added after the depolymerization of the paraformaldehyde in a basic medium, in order to form *N,N*-di(hydroxymethyl)aniline as an intermediate. Then, phloroglucinol was added at 0 °C, after which the product T-a slowly precipitated as it formed. Due to the *C*_3_ symmetry axis of T-a, the three *N*-phenyloxazine units are homotopic, making them equivalent and simplifying the ^1^H NMR spectrum. For this reason, all three oxazine rings appear as one and, therefore, T-a shows only two methylene signals, one for each type of methylene group (T-a, Figure 1b) [25].

According to these results, with more than one oxazine functionality in the benzene ring, the signals of the C_ar_C*H*_2_N group are shifted to higher fields (“b” signals, Figure 1b). This indicates that an increase on oxazine rings corresponds to an increase in electronic density on C_ar_C*H*_2_N, suggesting that the nitrogen basicity should also be higher. *N*,*O*-acetal signals appeared near 5.4 ppm in all cases. As they are situated further from the ring, the influence of more functionalities was limited (“a” signals, Figure 1b).

### 3.2. Polymerization and Thermal Stability of Polybenzoxazines

**Figure 2:**
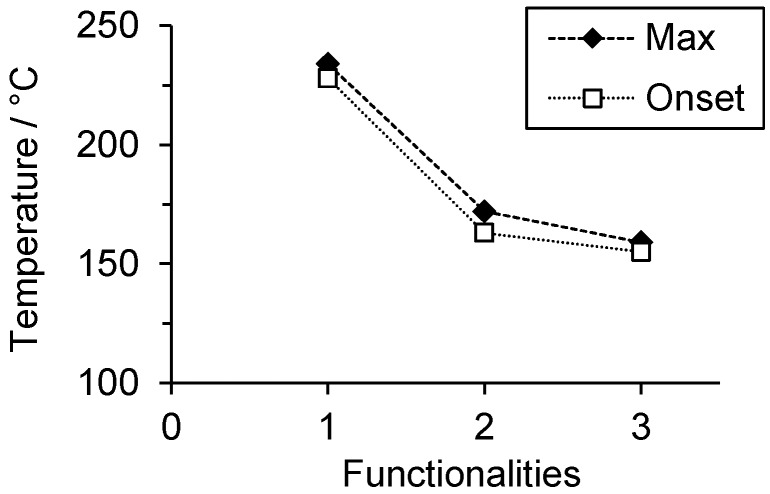
Exothermic polymerization peak (maximum and onset temperature) of P-a, R-a, and T-a, as determined by DSC, with a heating rate of 5 K/min.

In order to study the relative reactivity of the benzoxazines, the polymerization temperature was determined by DSC. P-a and R-a yielded polymerization temperatures at *T*_max_ of 234 and 172 °C, respectively, which is in accordance with previously reported studies [16]. The polymerization of T-a was observed at 159 °C. Thus, T-a polymerizes at even lower temperatures than R-a (Figure 2).

Monomer samples were polymerized by applying a stepwise temperature program to avoid the localized overheating of the sample upon exothermic curing.

The thermal stabilities of the polymers poly(P-a), poly(R-a) and poly(T-a) were determined by TGA in nitrogen. The temperatures of 5% and 10% weight loss values (*T*_5%_ and *T*_10%_) of both poly(R-a) and poly(T-a) were around 220 and 250 °C, respectively. In contrast, poly(P-a) showed higher *T*_5%_ and *T*_10%_ (237 and 281 °C) (Table 2). The results indicate a trend of improved thermal stability of poly(P-a) in contrast to polybenzoxazines based on di- or trifunctional benzoxazines.

**Figure 3:**
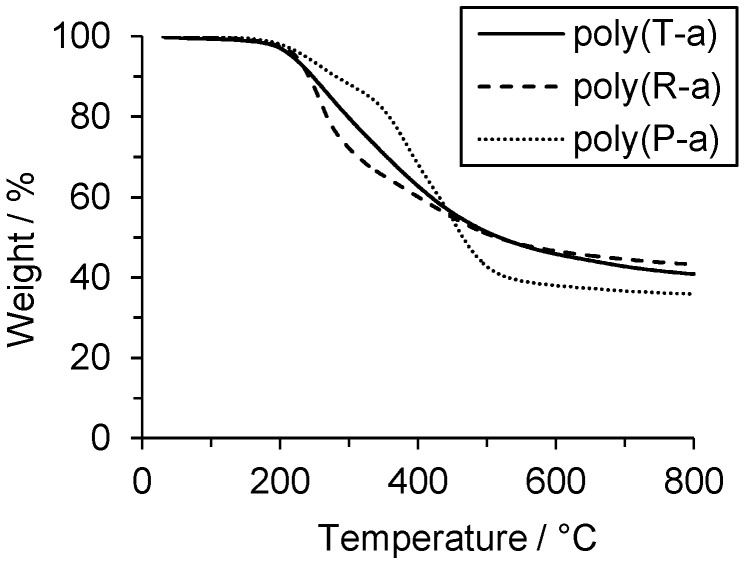
Thermogravimetric analysis of P-a, R-a, and T-a in nitrogen using a heating rate of 10 K/min.

Aromatic amine-based polybenzoxazines usually exhibit at least two decomposition events: one between 200 and 300 °C that corresponds to the amine elimination, and a second at around 400 °C due to phenolic cleavage [2,27]. Poly(P-a) showed an onset of the first weight loss step at 199 °C, a second one at 350 °C, and a third one at 442 °C. The final char yield at 800 °C was 36% of the initial weight. In the cases of poly(R-a) and poly(T-a), only one major decomposition step was observed. In case of poly(R-a), the first step was observed at 223 °C gaining a final char yield of 43% of the initial weight. The thermal decomposition of poly(T-a) started at 205 °C and continued to a final char yield of 41% at 800 °C. (Figure 3).

Poly(P-a), the polymer based in the monofunctional benzoxazine, showed the decomposition signal around 400 °C, which is assigned in the literature to phenolic cleavage. In contrast, that signal was absent in polymers derived from multifunctional benzoxazines R-a and T-a that, in addition, exhibited a higher char yield, probably due to a more cross-linked network. These differences in thermal behavior between poly(R-a) and poly(T-a), on the one hand, and poly(P-a), on the other hand, point towards different chemical structures.

### 3.3. Monomer and Polymer Characterization by IR

In order to gain insights into the resulting chemical structure of the polymers, IR spectra of the monomers and the corresponding polymers were obtained.

**Figure 4:**
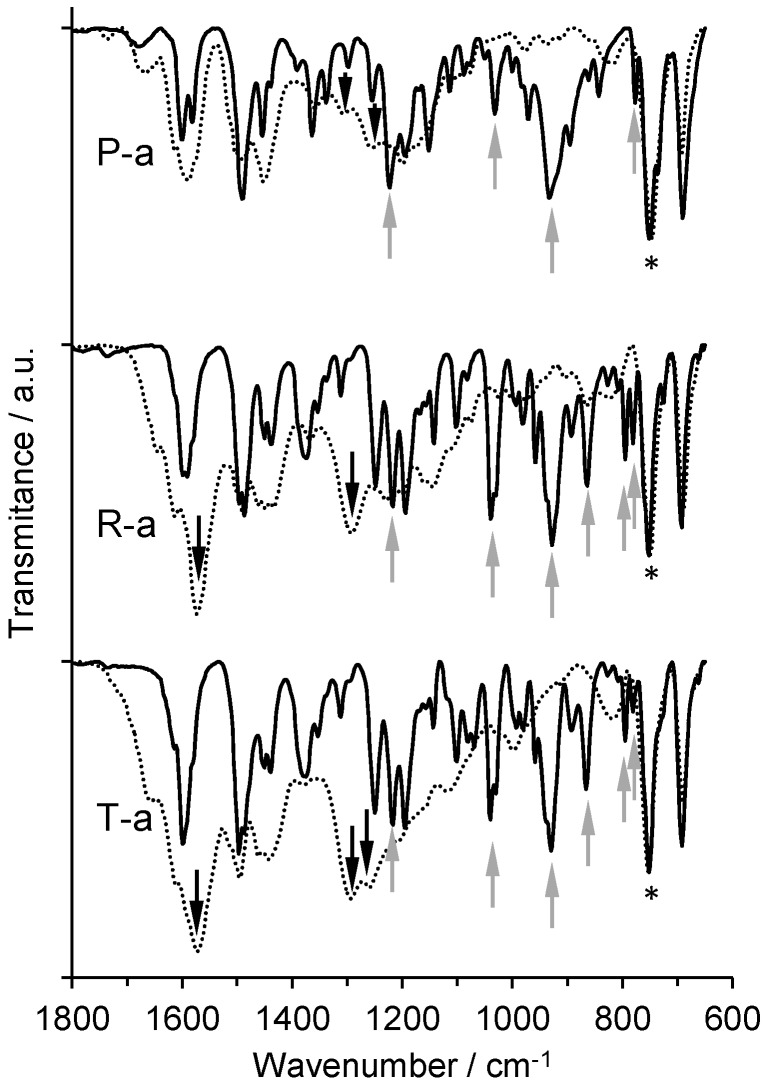
Infrared spectra of monomers P-a, R-a, and T-a (solid lines) and corresponding polymers (dotted lines). Signals that disappear in polymerization (grey up arrow), signals formed during polymerization (black down arrow), and the signal used as reference (asterisk) are indicated.

The signal at 752 cm^−1^ corresponds to C–N stretching in dialkyl aniline moiety, which was present in all monomers and polymers studied [28,29]. Therefore, this band was used to normalize the spectra. The pattern between 770 and 800 cm^−1^ (P-a: 777 cm^−1^, R-a: 781, 795 cm^−1^, T-a: 781, 795 cm^−1^) probably corresponds to out-of-plane C–H bending bands in the oxazine ring [24,30]. They disappeared after polymerization, which indicates a ring opening of the benzoxazine ring. The band at 930 cm^−1^ has been reported to represent different substitution patterns in benzene rings [16,31]. However, in our studies, that signal was present in all three monomers P-a, R-a, and T-a (P-a: 932 cm^−1^, R-a: 928 cm^−1^, T-a: 930 cm^−1^), each one exhibiting differently-substituted rings. As those bands were not present in the resulting polymers, they were assigned to out-of-plane vibrational modes of the oxazine rings in P-a, R-a, and T-a, respectively (Figure 4) [32].

Relevant information regarding polymer structure was obtained by studying the ether signals. Aromatic ether asymmetric stretching signals are usually strong bands in the range of 1200–1300 cm^−1^. In that region, however, there were several bands that could not be assigned unequivocally [28,29]. In return, the symmetric ether stretching was observed in all monomers (P-a: 1032 cm^−1^, R-a: 1038 cm^−1^, T-a: 1040 cm^−1^), but not in the appropriate polymers. That indicates that ether bridges are absent in the polymers, discarding the presence of phenoxy-type structures [16,30].

In all three cases, phenolic-type structures were further confirmed by the presence of C_ar_–OH stretching signals, observed at around 1300 cm^−1^ (poly(P-a): 1306 cm^−1^, poly(R-a): 1292 cm^−1^, poly(T-a): 1294 cm^−1^) and the signals of C_ar_OH···O intermolecular hydrogen bonding near 3370 cm^−1^ (poly(P-a): 3362 cm^−1^, poly(R-a): 3372 cm^−1^, poly(T-a): 3377 cm^−1^) [7].

In summary, complete ROP of benzoxazines occurred as a result of thermal curing of the monomers P-a, R-a, and T-a, leading to poly(P-a), poly(R-a) and poly(T-a) with phenolic-type structures.

### 3.4. Methylene Link Formation as Polymerization Pathway

**Scheme 2:**
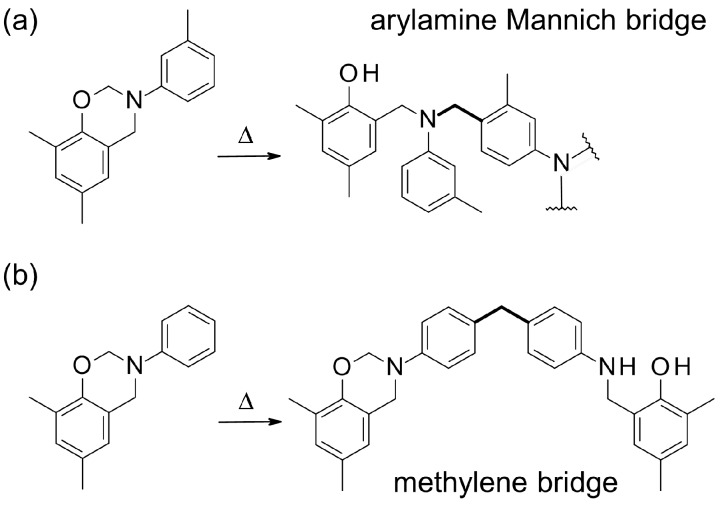
(**a**) Reaction of *N*-phenylbenzoxazine with phenolic *ortho* and *para* positions blocked and aniline activated in *meta*, as reported by Ishida et al. [4,33,34]; and (**b**) reaction of *N*-phenylbenzoxazine with phenolic *ortho* and *para* positions blocked, as described by Andreu et al. [1].

Phenolic-type structures in benzoxazines are formed by the nucleophilic attack of carbon atoms in *ortho* or *para* positions on the phenolic ring [2,13]. Alternatively, they can be formed by a rearrangement of phenoxy structures upon further heating. Both reaction pathways are valid for the mono- and bifunctional monomers P-a and R-a, but not for T-a, the trifunctional one, as its phenolic ring is completely substituted and, thus, has no reactive positions. *N*-phenylbenzoxazines with phenol nucleophilic *ortho* and *para* positions blocked could react by the *para* position of the aniline ring, if activated in *meta*, mainly forming arylamine Mannich bridges, as reported by Ishida et al. (Scheme 2a) [4,33,34]. In addition, when the *para* position of the aniline moiety was occupied, the ring-opening reaction occurred through the aniline *ortho* position according to Wang et al. [35]. Andreu et al. observed that *N*-phenylbenzoxazine with the *ortho* and *para* phenolic positions blocked could dimerize by linking aniline rings through methylene bridges (Scheme 2b) [1]. With respect to T-a, which exhibits a fully-blocked phenolic ring, the aniline moiety could react analogously, yielding a highly-branched polymer.

In order to test the possibility of benzoxazine polymerization through methylene bridges, each benzoxazine was reacted with *N*,*N*-dimethylaniline as a model dialkyl aniline ring [1]. The reactions were carried out with an excess of *N*,*N*-dimethylaniline (2 eq) in order to favor a benzoxazine/aniline reaction over benzoxazine self-polymerization. Reactions were performed for 60 min at 120 °C, after which the DMSO-*d*_6_ soluble parts were analyzed by ^1^H NMR.

**Scheme 3:**
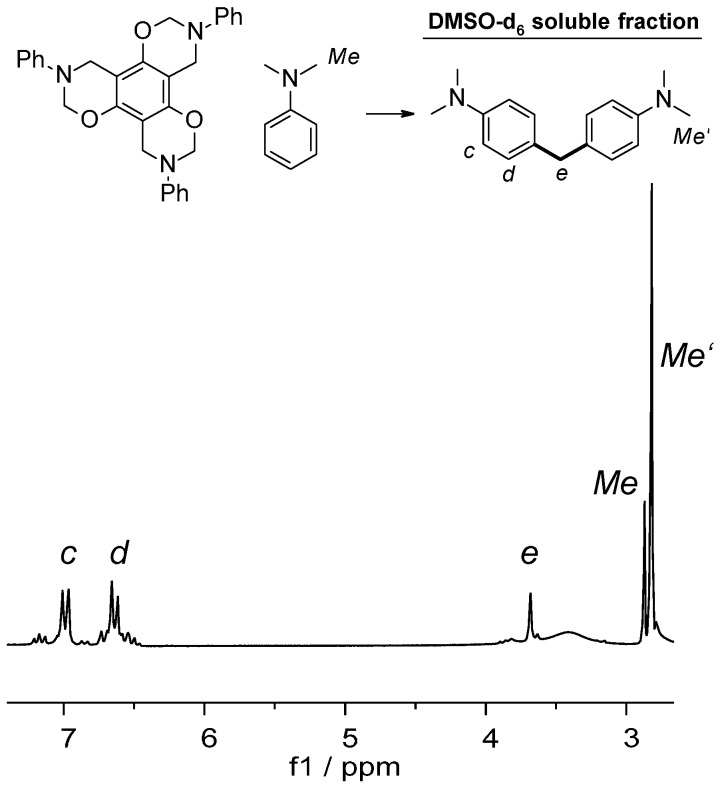
Formation of 4,4′-methylenebis-*N*,*N*-dimethylaniline in the reaction of T-a and *N*,*N*-dimethylaniline (120 °C/1 h) and the corresponding ^1^H NMR of DMSO-d_6_ soluble part.

With regard to the monofunctional P-a, both unreacted monomer and *N*,*N*-dimethylaniline were detected, indicating that neither a reaction nor a polymerization took place. However, the benzoxazines R-a and T-a, yielded no residual monomers after being reacted under those conditions. That observation indicated that they either reacted with the aniline or polymerized to non-soluble fractions. Regarding R-a, the soluble part of the reaction mixture contained unreacted *N*,*N*-dimethylaniline, indicating that polymerization took place. T-a’s reaction mixture, however, contained a mixture of *N*,*N*-dimethylaniline with 4,4′-methylenebis-*N*,*N*-dimethylaniline [26]. Formation of 4,4′-methylenebis-*N*,*N*-dimethylaniline was the result of connecting two *N*,*N*-dimethylaniline molecules by a methylene bridge in *para* position (Scheme 3).

When the same reaction of T-a was performed using 4,*N*,*N*-trimethylaniline with a blocked *para* position, exclusively 4,*N*,*N*,-trimethylaniline was detected in the reaction mixture, indicating that polymerization had still taken place, but that no coupling had occurred.

**Scheme 4:**
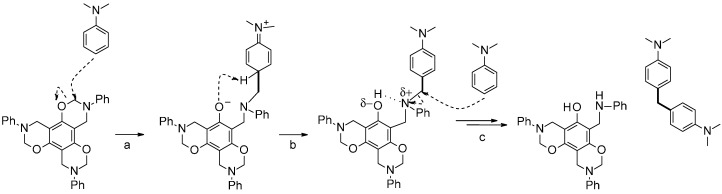
Proposed mechanism for the formation of 4,4′-methylenebis-*N*,*N*-dimethylaniline by thermal reaction of *N*,*N*-dimethylaniline with T-a. (**a**) Ring opening of the benzoxazine by nucleophilic attack of *para* carbon of *N,N*-dimethylaniline; (**b**) proton transfer and activation by hydrogen bond formation; and (**c**) nucleophilic attack, proton transfer to generate 4,4′-methylenebis-*N*,*N*-dimethylaniline.

Formally, the formation of 4,4′-methylenebis-*N*,*N*-dimethylaniline could be explained by a migration of a methylene group from an oxazine ring. A similar migration of methylene linkages has been reported for benzoxazine reactions performed in the presence of strong Lewis acids, such as BF_3_·OEt_2_ [12]. Alternatively, the formation of 4,4′-methylenebis-*N*,*N*-dimethylaniline could be seen as reaction analogous to dimerization of benzoxazine with substituted *ortho* and *para* positions (Scheme 2). The formation of such methylene linkages could proceed through a multi-step process implying interim formaldehyde equivalents [12]. The process could be described as depicted in Scheme 4. *N*,*N*-dimethylaniline opens the benzoxazine ring by a nucleophilic attack from the carbon *para* to the *N*,*O*-acetal. The hydrogen bond formed between the phenolic hydroxyl and amine group generates a partially positive charge on the nitrogen atom enhancing the electrophilicity of the methylene group in N*C*H_2_C_a_. A second *N*,*N*-dimethylaniline attacks that polarized carbon to yield the observed 4,4′-methylenebis(*N*,*N*-dimethylaniline).

Aniline moieties in the monomer T-a could behave analogously to *N*,*N*-dimethylaniline. Thus, the polymerization reaction of T-a could follow a similar course described in Scheme 4. If so, poly(T-a) should feature methylene bridges between aniline units in addition to the typical *N,O*-acetal or Mannich bridge. In order to confirm this hypothesis, the resulting polymers were analyzed using solid-state ^13^C NMR.

### 3.5. Structural Elucidation of Multifunctional Poly(benzoxazines)

Solid state ^13^C NMR was applied to describe the chemical structure of the multifunctional poly(benzoxazines). In a first step, a ^13^C CP experiment was recorded to evaluate the position of the spinning side bands in the spectrum. CP-TOSS experiments were recorded under the same conditions in order to suppress overlapping spinning side bands.

**Figure 5:**
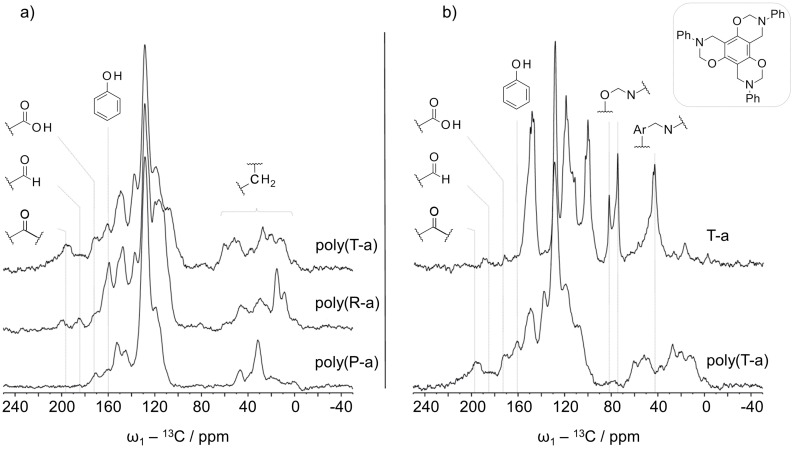
^13^C CP-TOSS experiments for: (**a**) polymers derived from P-a, R-a, and T-a and (**b**) comparison of T-a with poly T-a. All spectra were recorded under the same conditions (see experimental conditions).

The CP-TOSS spectra of polymers derived from P-a, R-a, and T-a are shown in Figure 5a. Peaks around 70–80 ppm corresponding to CH_2_ in *N*,*O*-acetal linkages were missing in all studied polymers indicating that all benzoxazine monomers have been opened completely and have rearranged to phenolic-type polybenzoxazines, which is in agreement with IR results (comparison T-a with poly(T-a), Figure 5b).

**Scheme 5:**
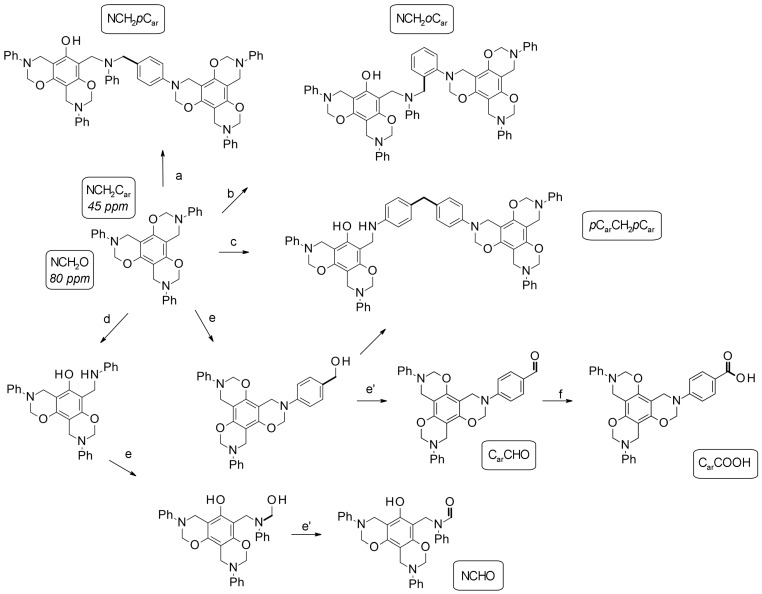
Possible polymerization reactions of trifunctional benzoxazine T-a. (**a**) Benzoxazine ring attacked by the para position of aniline; (**b**) benzoxazine ring attacked by the ortho position of aniline; (**c**) diphenyl methane bridge formation; (**d**) formaldehyde release; (**e**) and (**e’**) formylation using formaldehyde as the oxidant and reagent; and (**f**) oxidation.

With respect to poly(P-a) Mannich bridges can be formed between the nitrogen and an aromatic carbon from phenol or alternatively from the aniline. The appropriate methylene groups in poly(P-a) were aligned to peaks at 47 and 31 ppm. The signal was shifted up-field, which could be due to a stronger shielding of the groups at a polymeric stage. In comparison to poly(P-a), the aliphatic region of poly(R-a) and poly(T-a) was more complex indicating a variety of methylene linkages present in polymers derived from multifunctional benzoxazines. Furthermore, the higher oxazine functionality of R-a and T-a monomers entails the formation of different isomers leading to a more complicated polymer structure. In T-a and R-a, which have no or less free positions on the phenolic ring, respectively, the Mannich bridge could be formed between the nitrogen and the *para* or *ortho* positions of aniline moieties (Scheme 5a,b). With respect to the possible formation of diphenyl methane bridges in poly(T-a), signals observed around 60 ppm could represent that class of linkage. The connection by *ortho* and/or *para* positions would give rise to different diphenyl methane signals (Scheme 5c).

The complexity in the methylene region could be a consequence of different cross-linking via the postulated methylene migration processes. The chemical shift of a CH_2_-group in a diphenyl methane bridge; however, is very similar to the one in a Mannich bridge. Thus, signals of the diphenyl methane links could not been unambiguously assigned.

In addition to the expected aromatic peaks between 100 and 160 ppm, resonances at lower fields were observed. The spectra of all the polymers showed a signal at 170 ppm, which could be carboxylic acid or ester groups. The spectra of poly(R-a) and poly(T-a) exhibited additional signals typical for aldehyde (185 ppm) and ketone (200 ppm) functionalities, which were more prominent in the case of poly(T-a) (Figure 5a). The presence of groups with a higher oxidation state, such as carboxylic acids, aldehydes and ketones in the polymers might be related to the methylene migration process. A possible explanation would be the formation of free formaldehyde or formaldehyde equivalents in the reaction medium acting in methylene transfer processes while cross-linking. With a progressive polymerization, the mobility decreases promoting side-reactions of the formaldehyde equivalents, such as oxidation or formylation reactions yielding carboxylic acids, ketones, and aldehydes, respectively (Scheme 5e,f).

Taking into account all the experimental evidence, it is clear that multifunctional benzoxazines R-a and T-a enable polymerization pathways yielding more complex structures in comparison to conventional monofunctional benzoxazines.

## 4. Conclusions

In this paper, we have reported the synthesis of mono-, di-, and tri-functional benzoxazines using aniline, paraformaldehyde, phenol, resorcinol, and phloroglucinol, respectively. The polymerization temperatures of the mono-, di-, and trifunctional benzoxazines were studied with DSC, and showed a decreasing order with the monofunctional *N*-phenylbenzoxazine exhibiting the highest polymerization temperature and the trifunctional phloroglucinol-based benzoxazine showing the lowest curing temperature. It is suggested that the reactivity of *N*-phenylbenzoxazines is directly related to the number of oxazine functionalities present in the benzene ring. The polymer structure derived from the trifunctional benzoxazine was based on phenolic-type, but including carbonyl-derived structures as well. Based on the outcome of the reaction with *N*,*N*-dimethylaniline, a plausible mechanism for methylene linkages formation is proposed. To the best of our knowledge, this is the first example of trifunctional *N*-phenylbenzoxazine polymerization, which involves transfer of methylene groups. Those findings will open new possibilities for benzoxazine polymerization.

## Figures and Tables

**Table 1:** ^1^H-T_1_ time measurements for different benzoxazines.

Benzoxazine	^1^H T_1_/s
(R-a)	12.33
(T-a)	2.15
poly(P-a)	2.93
poly(R-a)	2.35
poly(T-a)	1.21

**Table 2:** 5% and 10% weight loss temperatures *T*_5%_ and *T*_10%_ determined by TGA (0 to 800 °C and a heating rate of 10 K/min).

Benzoxazine	*T*_5%_/°C	*T*_10%_/°C
poly(P-a)	237	281
poly(R-a)	223	241
poly(T-a)	214	245

## Supplementary Materials

The following are available online at www.mdpi.com/2073-4360/8/8/278/s1, Figure S1: ^1^H NMR of P-a; Figure S2: IR(ATR) of P-a; Figure S3: DSC of P-a; Figure S4: ^1^H NMR of R-a; Figure S5: IR(ATR) of R-a; Figure S6: DSC of R-a; Figure S7: ^1^H NMR of T-a; Figure S8: ^13^C NMR of T-a; Figure S9: IR(ATR) of T-a; Figure S10: DSC of T-a; Figure S11: IR of poly(P-a); Figure S12: IR of poly(R-a); Figure S13: IR of poly(T-a); Figure S14: ^13^C CP TOSS of poly(P-a); Figure S15: ^13^C CP TOSS of poly(R-a); Figure S16: ^13^C CP TOSS of poly(T-a). * signals detected in CPTOSS and not in solution ^13^C experiments.
