# Supplementary material for: Multifunctional Benzoxazines Feature Low Polymerization Temperature and Diverse Polymer Structures
Source: Polymers (Basel). 2016 Aug 2;8(8):278. doi: 10.3390/polym8080278 (PMC6432505; doi:10.3390/polym8080278)
Supplement: Supplementary file 1 [file polymers-08-00278-s001.pdf]

Supplementary Materials: Multifunctional Benzoxazines Feature Low Polymerization Temperature and Diverse Polymer Structures

Marc Soto, Matthias Hiller, Hartmut Oschkinat and Katharina Koschek

P-a

3-phenyl-2.4-dihydro-1.3-benzoxazine:

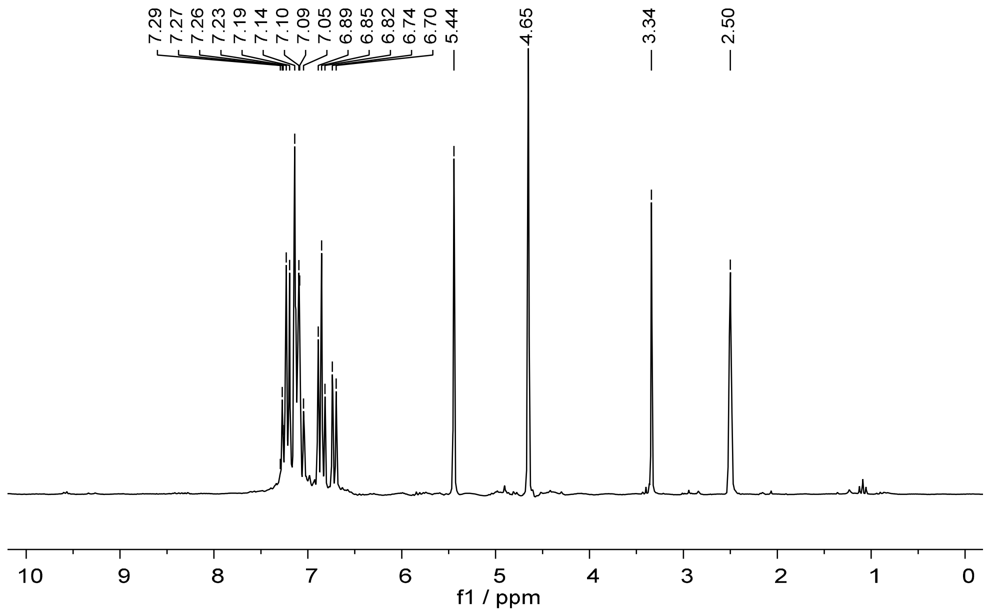

Figure S1. ^{1}H NMR of P-a.

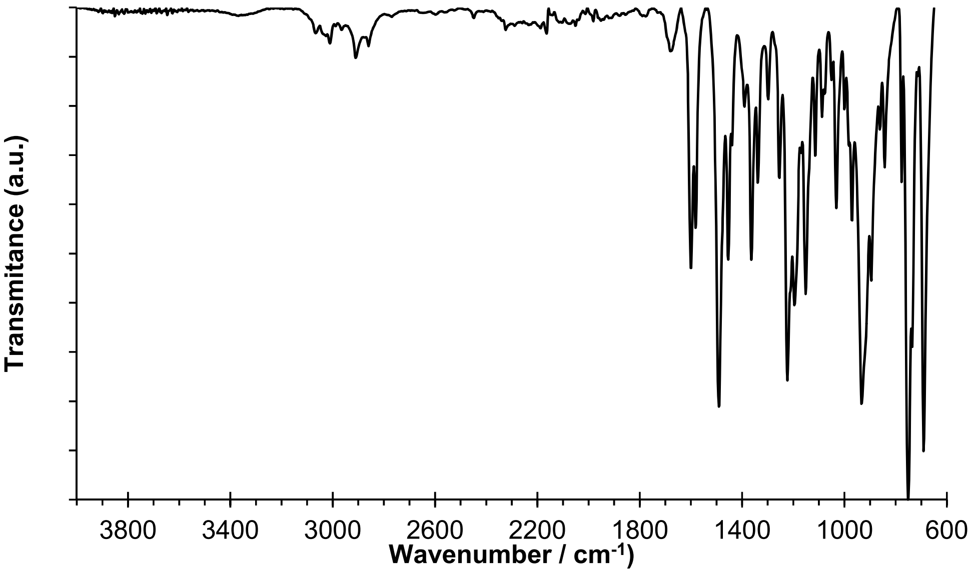

Figure S2. IR(ATR) of P-a.

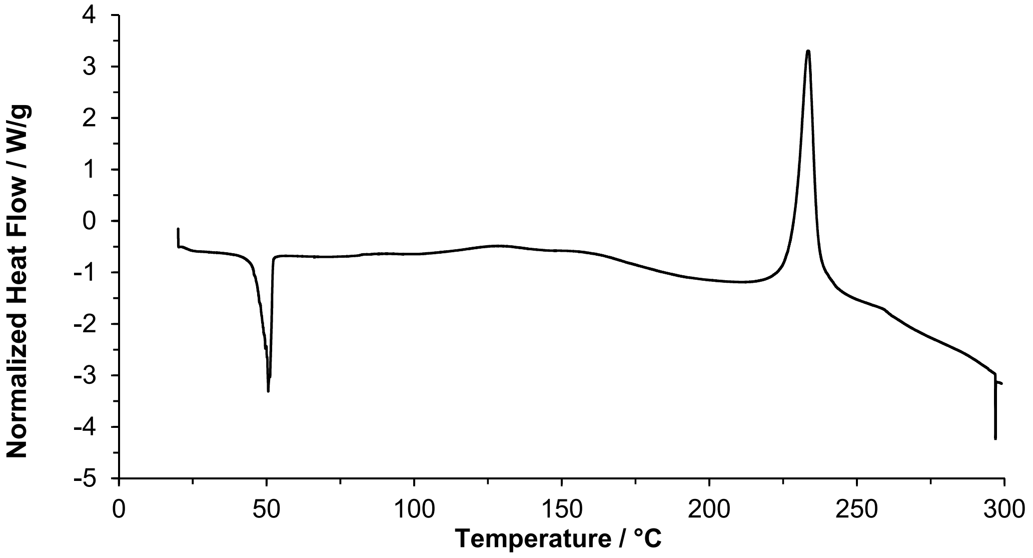

Figure S3. DSC of P-a.

R-a

3,9-diphenyl-2,4,8,10-tetrahydro-[1,3]oxazino[6,5-f][1,3]benzoxazine:

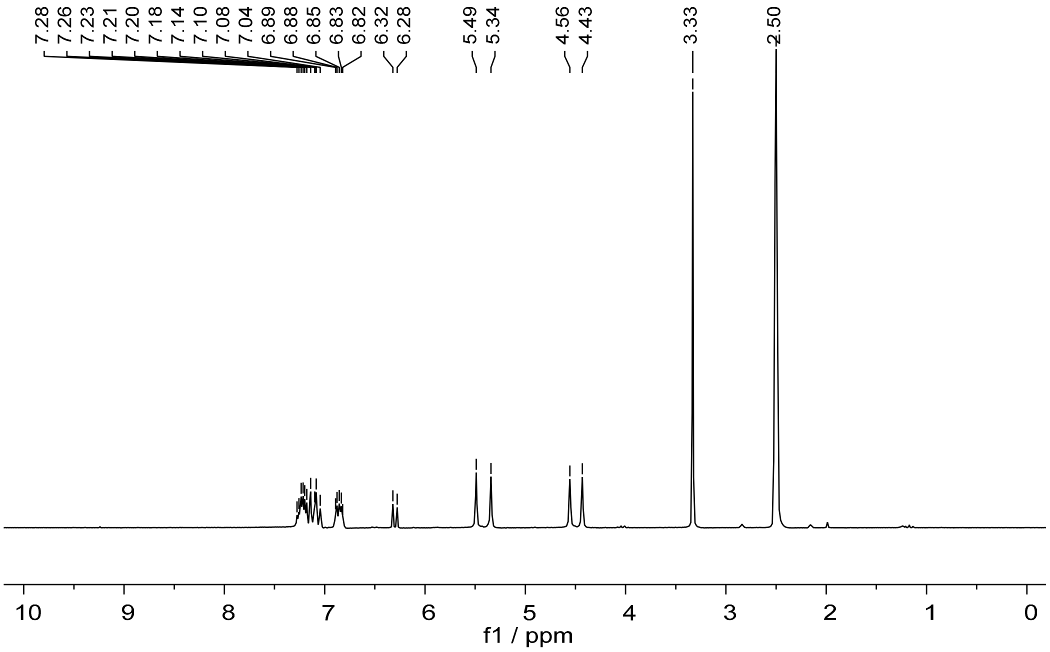

Figure S4. ^{1}H NMR of R-a.

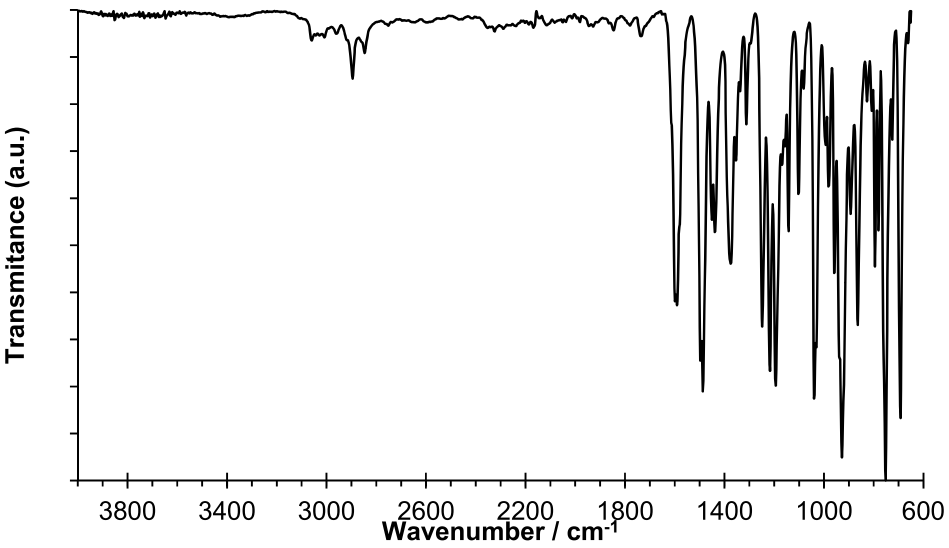

Figure S5. IR(ATR) of R-a.

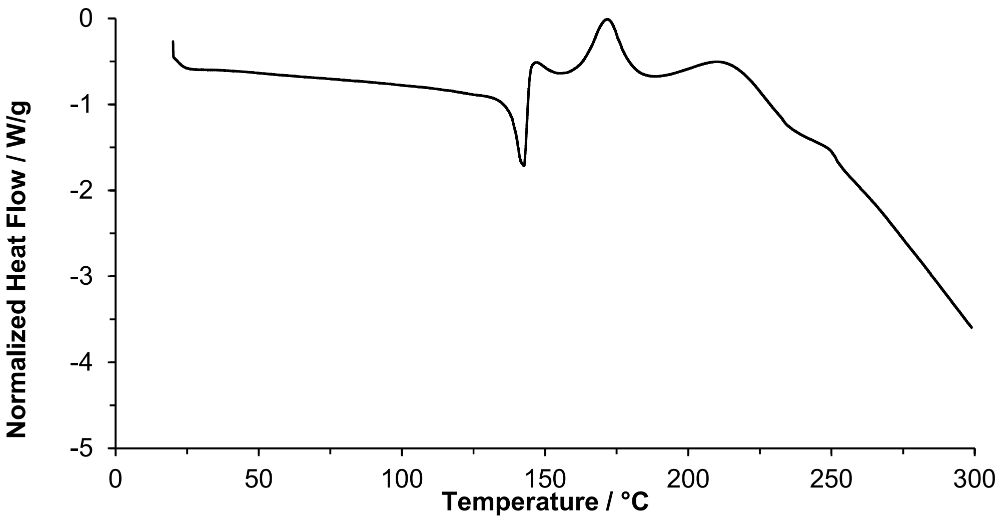

Figure S6. DSC of R-a.

T-a

3,7,11-triphenyl-3,4,6,7,8,10,11,12-octahydro-2H-1,5,9-trioxa-3,7,11-triazatriphenylene:

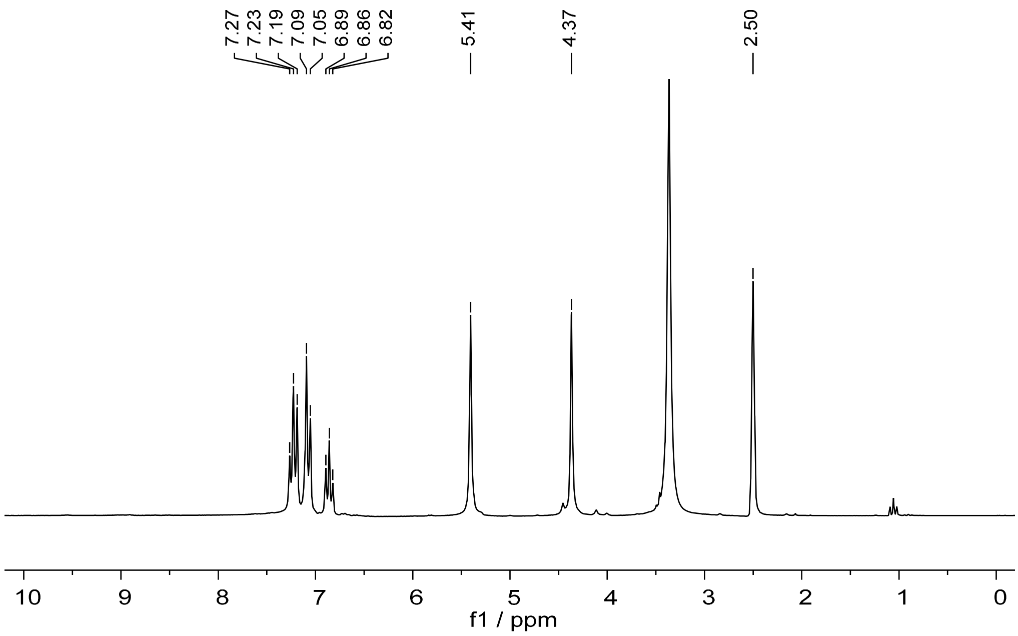

Figure S7. ^{1}H NMR of T-a.

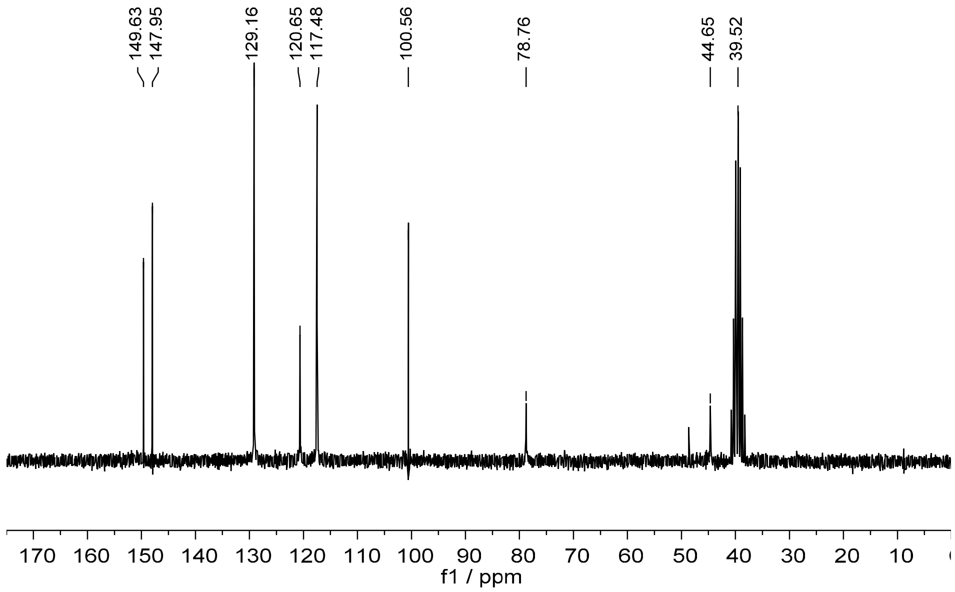

Figure S8. ^{13}C NMR of T-a.

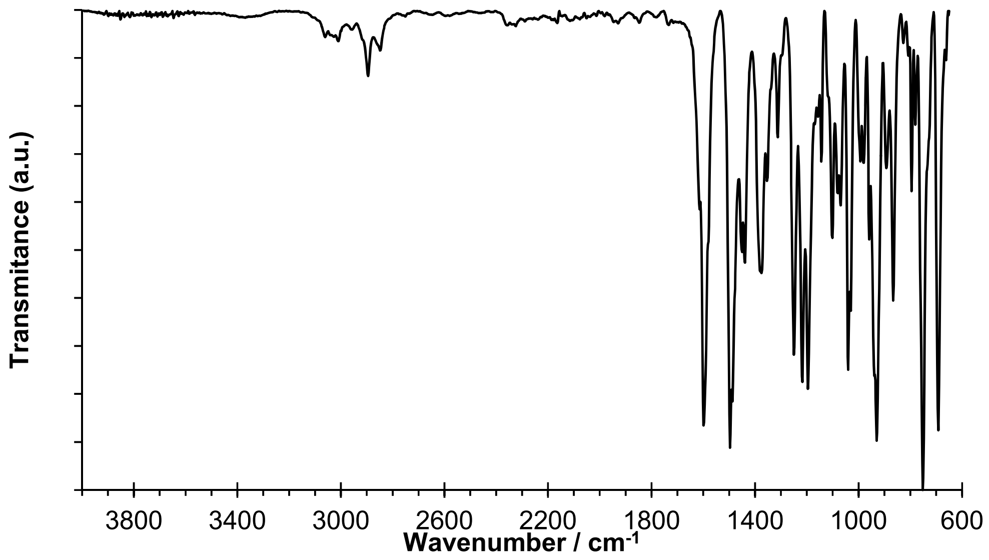

Figure S9. IR(ATR) of T-a.

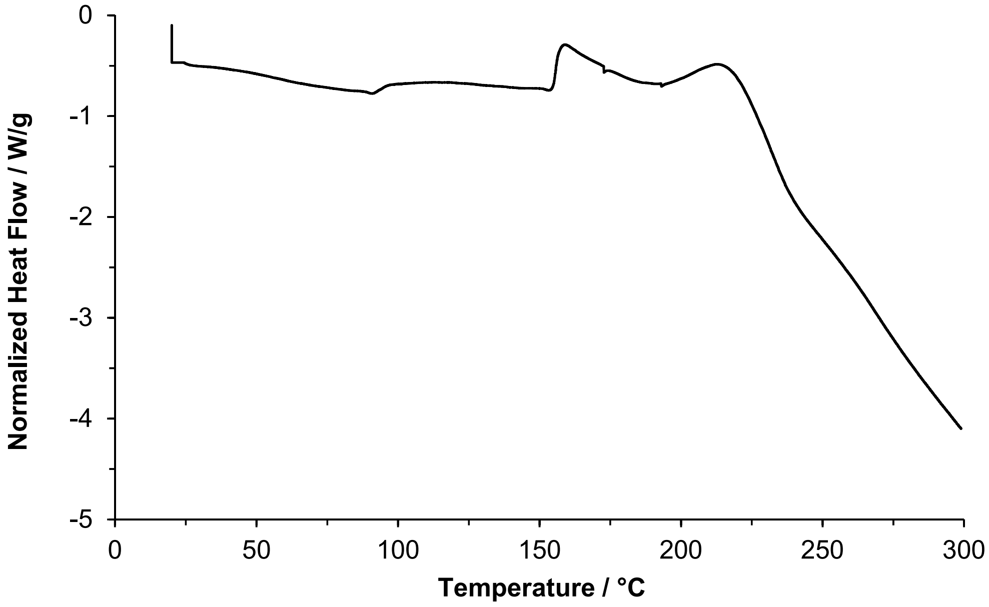

Figure S10. DSC of T-a.

IR of polymers

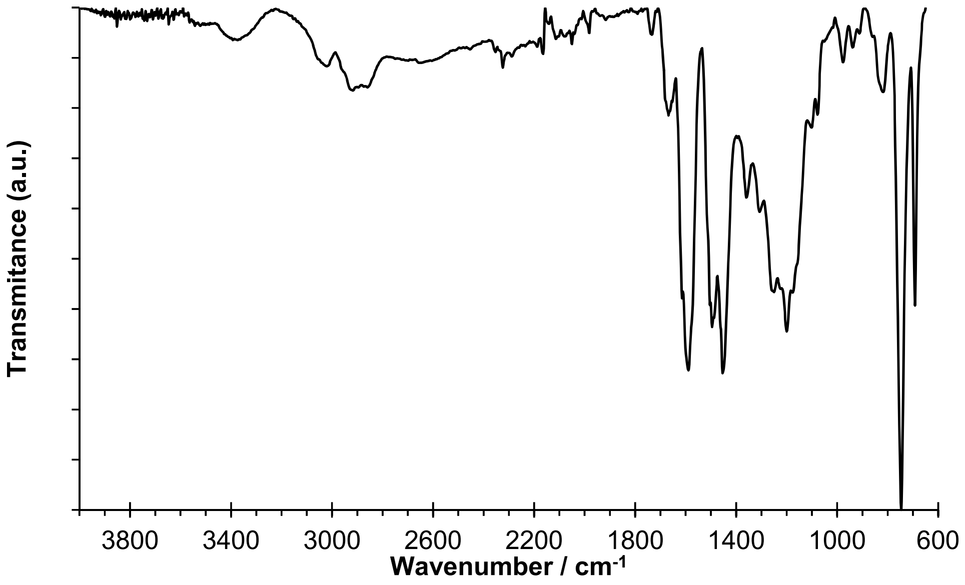

Figure S11. IR of poly(P-a).

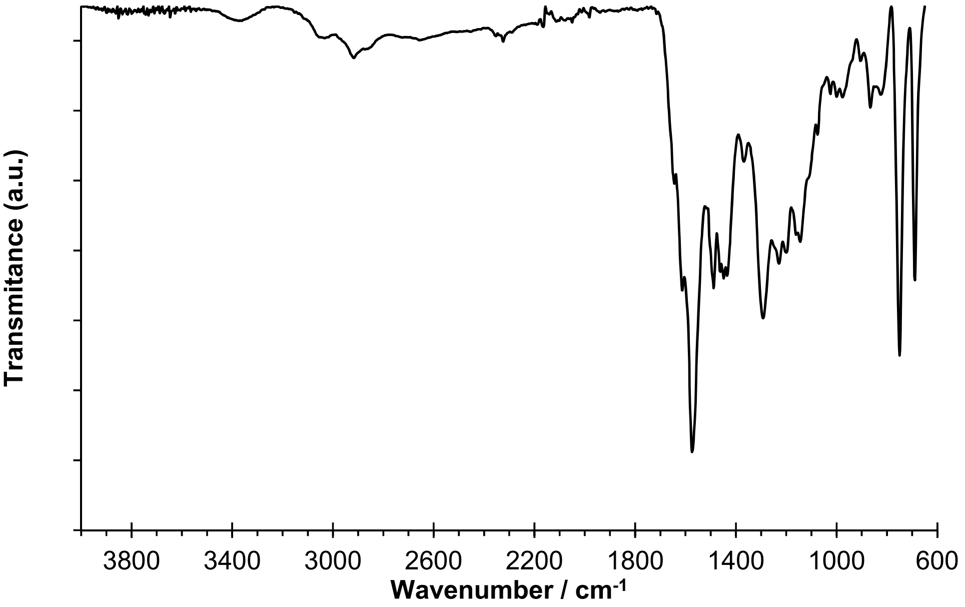

Figure S12. IR of poly(R-a).

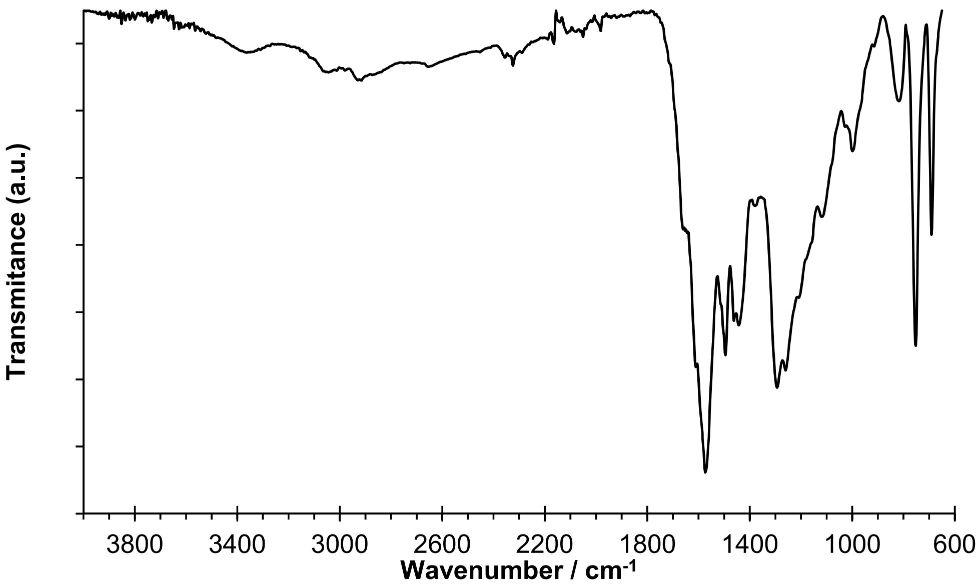

Figure S13. IR of poly(T-a).

^{13}C CPTOSS experiments of polymers

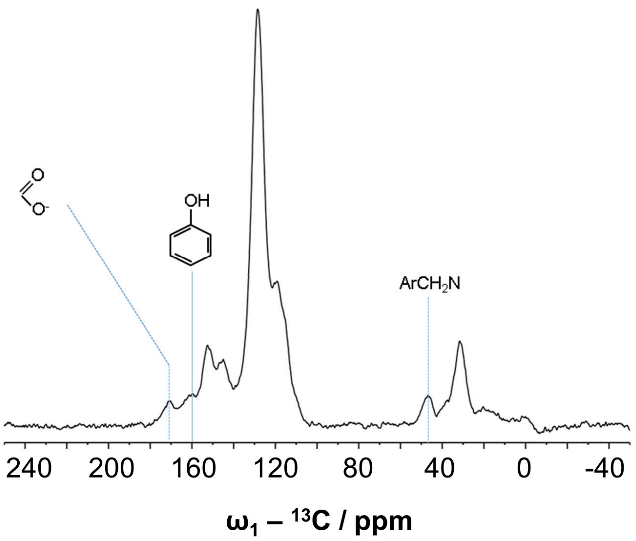

Figure S14. ^{13}C CP TOSS of poly(P-a).

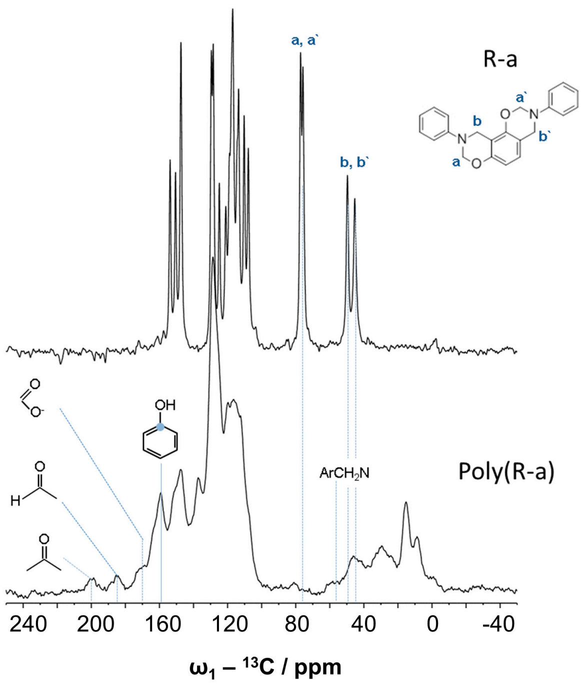

Figure S15. ^{13}C CP TOSS of poly(R-a).

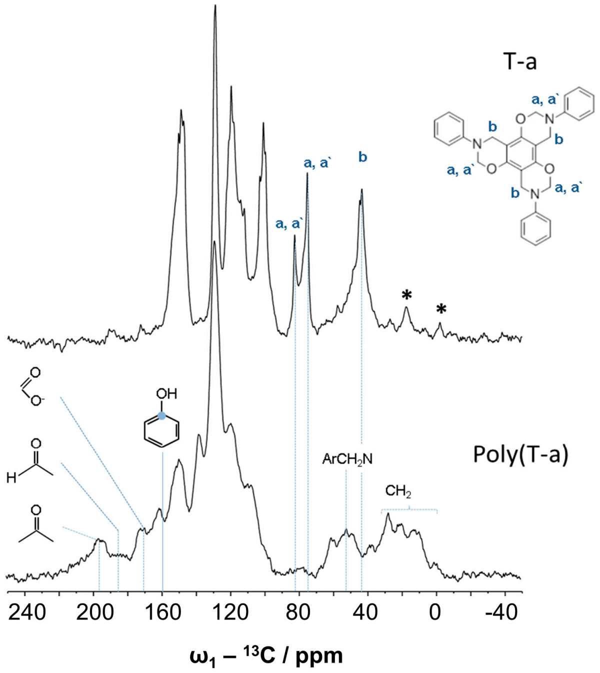

Figure S16. ^{13}C CP TOSS of poly(T-a). * signals detected in CPTOSS and not in solution ^{13}C experiments.

© 2016 by the authors; licensee MDPI, Basel, Switzerland. This article is an open access article distributed under the terms and conditions of the Creative Commons Attribution (CC-BY) license (http://creativecommons.org/licenses/by/4.0/).
